# Association between long-term smoking cessation and COVID-19 outcomes: Findings from a nationwide crosssectional online survey in China

**DOI:** 10.18332/tid/209212

**Published:** 2025-09-26

**Authors:** Xinmei Zhou, Ailifeire Aihemaiti, Anqi Cheng, Zhao Liu, Zheng Su, Ying Xie, Zhenxiao Huang, Liang Zhao, Xin Xia, Yi Liu, Qingqing Song, Dan Xiao, Chen Wang

**Affiliations:** 1 Department of Tobacco Control and Prevention of Respiratory Diseases, Center of Respiratory Medicine, China-Japan Friendship Hospital, Beijing, China; 2 National Center for Respiratory Medicine, Beijing, China; 3 State Key Laboratory of Respiratory Health and Multimorbidity, Beijing, China; 4 National Clinical Research Center for Respiratory Diseases, Beijing, China; 5 Institute of Respiratory Medicine, Chinese Academy of Medical Sciences, Beijing, China; 6 China-Japan Friendship Hospital (Institute of Clinical Medical Sciences), Chinese Academy of Medical Sciences & Peking Union Medical College, Beijing, China; 7 School of Health Policy and Management, Chinese Academy of Medical Sciences and Peking Union Medical College, Beijing, China; 8 School of Population Medicine and Public Health, Chinese Academy of Medical Sciences and Peking Union Medical College, Beijing, China; 9 China-Japan Friendship School of Clinical Medicine, Capital Medical University, Beijing, China; 10 Chinese Academy of Medical Sciences and Peking Union Medical College, Beijing, China

**Keywords:** smoking cessation, COVID-19, pneumonia, infection

## Abstract

**INTRODUCTION:**

Smoking has been identified as a potential risk factor for adverse COVID-19 outcomes. This study aimed to investigate the association between long-term smoking cessation and COVID-19 outcomes.

**METHODS:**

In this nationwide, cross-sectional online survey conducted in China (January–February 2023), 22709 adults with COVID-19, confirmed by nucleic acid amplification test (NAAT) or SARS-CoV-2 antigen testing, were included. Smoking status was self-reported and classified as never smokers, long-term ex-smokers (≥10 years), ex-smokers (<10 years), and current smokers. COVID-19 outcomes, including pneumonia, hospitalization, and severe COVID-19, were compared across these groups. Logistic regression models were used to adjust for potential confounders. Sensitivity analyses included all self-reported cases irrespective of test confirmation.

**RESULTS:**

Among 22709 COVID-19-positive participants, current smokers and ex-smokers <10 years exhibited significantly higher proportion of pneumonia, hospitalization, and severe COVID-19 than never smokers. Current smokers (AOR=3.18; 95% CI: 2.90–3.48) and ex-smokers quit <10 years (AOR=3.48; 95% CI: 2.96–4.09) had increased odds of pneumonia, whereas long-term ex-smokers showed no elevated risk (AOR=1.12; 95% CI: 0.45–2.41). These associations were consistent in sensitivity analyses. Other factors significantly associated with pneumonia included sex, education level, residence, obesity, income, and chronic conditions.

**CONCLUSIONS:**

Long-term smoking cessation was not associated with an elevated risk of COVID-19-related pneumonia compared to never smokers, whereas ex-smokers (<10 years) and current smokers remained high-risk groups. These findings support the potential benefits of sustained cessation, although further longitudinal studies are needed to confirm and extend these findings.

## INTRODUCTION

The COVID-19 pandemic has triggered a global health crisis^1^, compelling extensive research into identifying risk factors that influence infection susceptibility and outcomes^2^. Among these risk factors, smoking has emerged as a critical area of investigation, given its well-established role for chronic respiratory diseases, cancers, cardiovascular diseases and diabetes^3^. During the pandemic, the role of smoking as a potential risk factor for infection and adverse COVID-19 outcomes has garnered substantial attention^4^, with studies indicating elevated risks of hospital admission, disease severity progression, and mortality among individuals with smoking history^5,6^.

The relationship between smoking cessation and health outcomes has been well-documented across various diseases. Quitting smoking represents a transformative juncture in the trajectory of an individual’s health, markedly reducing the incidence and mortality rates of smoking-related diseases^7^. Smoking cessation diminishes the risk of chronic obstructive pulmonary disease (COPD) and asthma^8,9^. Furthermore, quitters had a slower decline in pulmonary function than continuous smokers^10^. Smoking cessation also confers a reduction in the incidence of cancers such as lung cancer. Remarkably, as the duration of smoking abstinence lengthens, the risk of developing these malignant neoplasms gradually diminishes^7^. A similar trend is observed for cardiovascular diseases, including coronary heart disease and stroke^11,12^. While short-term smoking cessation may temporarily elevate the risk of diabetes due to weight gain, long-term smoking cessation precipitates a gradual reduction^13,14^.

Despite the well-documented benefits of smoking cessation on overall health, a notable gap in knowledge persists regarding the relationship between long-term smoking cessation and COVID-19. Most studies pertaining to COVID-19 often categorize smoking merely as a binary factor – ever smoking or never smoking – or use classifications such as current smokers, ex-smokers, and never smokers^5^. These approaches do not sufficiently capture the effects of smoking cessation over time, particularly the potential benefits of long-term cessation. The effects of long-term smoking cessation on COVID-19 outcomes remain an area warranting more comprehensive investigation. We therefore hypothesized that the risk of adverse COVID-19 outcomes declines with longer duration of smoking cessation.

To address the critical knowledge gap, we conducted a nationwide cross-sectional study in China during the major COVID-19 outbreak of early 2023^15^, investigating the association between smoking status and COVID-19 outcomes, with a novel focus on long-term smoking cessation.

## METHODS

### Study population

**Figure 1:**
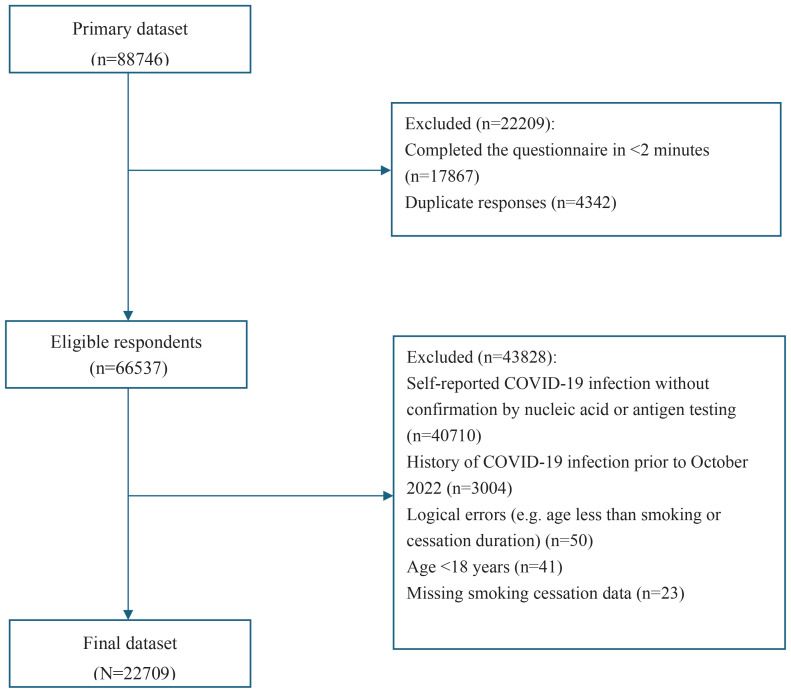
Flow chart of participant enrollment and exclusion from a nationwide cross-sectional online survey in China, Oct 2022–Feb 2023

This cross-sectional study utilized data from a nationwide online survey conducted in China (January–February 2023) following STROBE guidelines. This study was approved by the Review Board of China-Japan Friendship Hospital (2022-KY-183-1). A total of 88746 individuals initially completed the online questionnaire (Figure 1). After initial quality control measures, 22209 participants were excluded, comprising those who completed the questionnaire in less than 2 minutes (n=17867) and duplicate responses (n=4342). This left 66537 eligible respondents. From this group, an additional 43828 participants were excluded due to: self-reported COVID-19 infection without nucleic acid amplification test (NAAT) or SARS-CoV-2 antigen testing confirmation (n=40710); a history of COVID-19 infection prior to October 2022 (n=3004); logical errors (e.g. age less than smoking or cessation duration, n=50); aged <18 years (n=41); and missing smoking cessation data (n=23). This rigorous exclusion process resulted in a final analytic dataset of 22709 participants.

### Data collection

The recruitment of participants was conducted through the utilization of the web-based survey platform ‘Questionnaire Star’, which facilitated the collection of detailed information on participants’ demographics, smoking history, COVID-19 infection status, COVID-19 symptoms, and COVID-19 outcomes. Informed consent was obtained from all participants as an integral part of the study. Detailed recruitment information can be found in a previous article^16^.

### Definition of smoking status and potential confounders

Participants self-reported smoking status, categorized as current smokers, ex-smokers and never smokers. Within the group of ex-smokers, a further stratification was applied based on the duration of smoking cessation, utilizing a 10-year threshold^17^, resulting in two subgroups: ex-smokers (<10 years) and long-term ex-smokers (quit ≥10 years). Obesity was categorized as a binary variable: individuals with a body mass index (BMI) of ≥30 kg/m^2^ were classified as obese, while others were considered not obese^18^. Vaccination status was self-reported by participants indicating whether they had received a COVID-19 vaccine. Other covariates included age, sex (male, female), education level, residence (urban, rural), and income (<1000, 1000–2999, 3000–5999, 6000–9999, and ≥10000 RMB) (1000 Chinese Renminbi about US$140).

### Definition of COVID-19-related outcomes

Pneumonia encompassed individuals who sought medical attention due to a COVID-19 infection and received a diagnosis of pneumonia during their medical visit. COVID-19 hospital admission applied when individuals sought medical attention at healthcare facilities due to COVID-19 and required admission to the hospital. Severe COVID-19 was defined as cases in which participants sought medical attention due to a COVID-19 infection and reported being diagnosed as severe or critical.

### Statistical analysis

Descriptive statistics were compared using analysis of variance (ANOVA) or Kruskal-Wallis tests for continuous variables and chi-squared tests or Fisher’s exact probability tests for categorical variables. To further compare differences between smoking status groups, *post hoc* pairwise analyses were conducted following a significant overall test. Bonferroni correction was applied to control for Type I errors, with the adjusted significance level set at α=0.0083. Logistic regression models were performed to evaluate the associations between smoking status and COVID-19 outcomes, with a particular focus on pneumonia, which showed significant associations with long-term smoking cessation. The analysis focused on outcomes where significant differences were identified between ex-smokers quit <10 years and long-term ex-smokers (≥10 years) in exploratory analyses. This targeted approach reflects the primary research objective of assessing the impact of long-term smoking cessation on COVID-19-related outcomes. The results were expressed as adjusted odds ratios (AORs) with 95% confidence intervals (CIs). Models were adjusted for a predefined set of covariates based on existing literature, including age, sex, urban or rural residence, income, vaccination status, obesity, and chronic conditions (malignant tumors, cardiovascular diseases, chronic respiratory diseases, and diabetes). Adjusted absolute risks of pneumonia across smoking statuses were calculated using the *LSMEANS* statement within the PROC LOGISTIC procedure in SAS (version 9.4). In the logistic regression model. In cases where data pertaining to age (n=1814) were absent, a rigorous imputation process was applied employing multiple imputation techniques facilitated by the R package *MASS*. Individuals with missing smoking cessation data were excluded (n=23). Analyses were performed using SAS statistical software version 9.4 (SAS Institute).

### Patient and public involvement statement

Patients or the public were not involved in the design, conduct, or reporting of this study. This study was conducted during the COVID-19 pandemic, and the questionnaire was distributed online to facilitate rapid data collection. Participants were encouraged to share the survey link with their social networks to maximize the sample size; however, they were not involved in the research design or decision-making process.

## RESULTS

### Participant characteristics

Out of an initial 88746 participants, 22709 met the inclusion criteria and were analyzed (Figure 1). Among the 22709 participants included in the analysis (Table 1), 56.1% were male, with a mean age of 40.94 years (SD=11.63) and a mean BMI of 21.93 kg/m^2^ (SD=4.06). In terms of smoking status, 22.48% were current smokers, 3.96% were ex-smokers (<10 years), and 0.22% had quit for ≥10 years (n=51). The majority of participants (73.33%) reported never smoking. Significant overall differences were observed across smoking status groups in terms of gender, education level, residence, BMI, income, vaccination status, and comorbidities (p<0.0001 for each variable, except residence p=0.0005). Current smokers and ex-smokers (<10 years) were predominantly male, whereas only 48.5% of never smokers were male. The proportion of participants with a Bachelor’s degree or higher was highest among never smokers (65.53%). Never smokers consistently had the lowest proportions of cardiovascular diseases, diabetes, chronic respiratory diseases, and malignant tumors. The proportion of participants with cardiovascular diseases was higher among ex-smokers (<10 years, 25.67%; ≥10 years, 25.49%) and current smokers (23.51%) compared to never smokers (13.77%). The proportion of chronic respiratory diseases among ex-smokers (<10 years) (26.67%) and current smokers (23.8%) was approximately three times that of never smokers (8.36%). The proportion of malignant tumors was also higher in both current (5%) and ex-smokers (<10 years, 6.67%; ≥10 years, 5.88%) than in never smokers (1.53%). The proportion of participants who received COVID-19 vaccination was lowest in the never smoker group (84.19%).

**Table 1:** Demographic characteristics of study participants by smoking status from a nationwide crosssectional online survey in China, Oct 2022–Feb 2023 (N=22709)^a^

*Characteristics*	*Total* *(N=22709)* *n (%)*	*Current* *smokers* *(N=5105)* *n (%)*	*Ex-smokers <10* *years* *(N=900)* *n (%)*	*Long-term ex-smokers* *(N=51)* *n (%)*	*Never smokers* *(N=16653)* *n (%)*	*p*
**Sex**						<0.0001
Male	12740 (56.1)	3954 (77.45)	656 (72.89)	46 (90.2)	8084 (48.54)	
Female	9969 (43.9)	1151 (22.55)	244 (27.11)	5 (9.8)	8569 (51.46)	
**Age** (years), mean ± SD	40.94 ± 11.63	40.81 ± 11.85	41.17 ± 11.88	42.47 ± 9.82	40.96 ± 11.56	0.2616
**BMI**, mean ± SD	21.93 ± 4.06	22.10 ± 4.17	21.73 ± 4.18	23.11 ± 4.34	21.88 ± 4.01	<0.0001
**Residence**						0.0005
City	16442 (72.4)	3809 (74.61)	631 (70.11)	38 (74.51)	11964 (71.84)	
Rural	6267 (27.6)	1296 (25.39)	269 (29.89)	13 (25.49)	4689 (28.16)	
**Education level**						<0.0001
Middle school or lower	3611 (15.9)	1098 (21.51)	202 (22.44)	12 (23.53)	2299 (13.81)	
High school/vocational	5112 (22.51)	1394 (27.31)	266 (29.56)	11 (21.57)	3441 (20.66)	
Bachelor’s or higher	13986 (61.59)	2613 (51.19)	432 (48)	28 (54.9)	10913 (65.53)	
**Income** (RMB)						<0.0001
<1000	2125 (9.36)	318 (6.23)	54 (6)	5 (9.8)	1748 (10.5)	
1000–2999	4412 (19.43)	1127 (22.08)	184 (20.44)	8 (15.69)	3093 (18.57)	
3000–5999	8696 (38.29)	2136 (41.84)	368 (40.89)	21 (41.18)	6171 (37.06)	
6000–9999	5280 (23.25)	1110 (21.74)	238 (26.44)	13 (25.49)	3919 (23.53)	
≥10000	2196 (9.67)	414 (8.11)	56 (6.22)	4 (7.84)	1722 (10.34)	
**Comorbidities^b^**						
Cardiovascular disease	3128 (13.77)	1200 (23.51)	231 (25.67)	13 (25.49)	1684 (10.11)	<0.0001
Diabetes	2537 (11.17)	1132 (22.17)	222 (24.67)	6 (11.76)	1177 (7.07)	<0.0001
Chronic respiratory disease	2857 (12.58)	1215 (23.8)	240 (26.67)	9 (17.65)	1393 (8.36)	<0.0001
Malignant neoplasm	573 (2.52)	255 (5)	60 (6.67)	3 (5.88)	255 (1.53)	<0.0001
None of the above chronic diseases	15448 (68.03)	2149 (42.1)	320 (35.56)	27 (52.94)	12952 (77.78)	<0.0001
**Vaccination status**						<0.0001
Yes	19656 (86.56)	4785 (93.73)	807 (89.67)	44 (86.27)	14020 (84.19)	

a Comparisons across smoking status were conducted using analysis of variance (ANOVA) or Kruskal–Wallis tests for continuous variables, and chi-squared or Fisher’s exact tests for categorical variables, as appropriate.

b The sum of percentages for the four chronic disease categories (cardiovascular disease, diabetes, chronic respiratory disease, malignant neoplasm) and the ‘None of the above’ category may exceed 100% because participants with comorbid conditions are counted in multiple disease categories. BMI: body mass index (kg/m^2^). RMB: 1000 Chinese Renminbi about US$140.

### Distribution of COVID-19 outcomes by smoking status

The proportions of pneumonia, hospitalization, and severe COVID-19 are presented in Table 2. Among the study population, pneumonia occurred in 14.40% of participants, 5.83% required hospitalization, and 1.71% developed severe COVID-19. Pneumonia was observed in >30% of both current smokers and ex-smokers (<10 years), with no statistically significant difference between these groups (p=0.0877). Current smokers showed approximately four times the proportion of pneumonia compared with never smokers (p<0.0001). Both current smokers and ex-smokers (<10 years) had significantly higher proportions than long-term ex-smokers (13.73%; p=0.0076 and p=0.0031, respectively). Although the proportion in long-term ex-smokers was higher than in never smokers, this difference was not statistically significant (p=0.1266).

**Table 2:** Proportions of COVID-19-related outcomes (pneumonia, hospitalization, and severe COVID-19) by smoking status from a nationwide cross-sectional online survey in China, Oct 2022–Feb 2023 (N=22709)

*Outcomes*	*Total* *n (%)*	*Current smokers* *n (%)*	*Ex-smokers <10* *years* *n (%)*	*Long-term ex-smokers* *n (%)*	*Never smokers* *n (%)*	*p ^a^*
**Pneumonia^b^**						<0.001
Yes	3270 (14.4)	1639 (32.11)	315 (35)	7 (13.73)	1309 (7.86)	
No	19439 (85.6)	3466 (67.89)	585 (65)	44 (86.27)	15344 (92.14)	
**Hospitalization^c^**						<0.001
Yes	1323 (5.83)	481 (9.42)	89 (9.89)	2 (3.92)	751 (4.51)	
No	21386 (94.17)	4624 (90.58)	811 (90.11)	49 (96.08)	15902 (95.49)	
**Severe COVID-19^d^**						<0.001
Yes	388 (1.71)	173 (3.39)	29 (3.22)	1 (1.96)	185 (1.11)	
No	22321 (98.29)	4932 (96.61)	871 (96.78)	50 (98.04)	16468 (98.89)	

a Statistical comparisons employed chi-squared tests for pneumonia outcomes and Fisher’s exact probability tests for hospitalization and severe COVID-19 outcomes.

b Pneumonia: defined as individuals who sought medical attention due to a COVID-19 infection and received a diagnosis of pneumonia during the medical visit.

c Hospitalization: defined as individuals who sought medical attention at healthcare facilities due to COVID-19 and required hospital admission.

d Severe COVID-19: defined as individuals who sought medical attention due to a COVID-19 infection and reported being diagnosed as severe or critical.

For hospitalization, proportions among current smokers (9.42%) and ex-smokers (cessation <10 years, 9.89%) both exceeded 9%, with no statistically significant difference between these groups (p= 0.6595). Long-term ex-smokers (3.92%) and never smokers (4.51%) had similar proportions at approximately 4%. Owing to the limited number of hospitalizations in long-term ex-smokers (n=2), comparisons with this group showed no statistically significant differences (vs current smokers: p=0.1957; vs ex-smokers <10 years: p=0.1754). However, both current smokers and ex-smokers (<10 years) had significantly higher proportions of hospitalization than never smokers (both p<0.0001).

Current smokers (3.39%) and ex-smokers <10 years (3.22%) had proportions of severe COVID-19 approximately three times higher than never smokers (both p<0.0001). Both groups also showed higher proportions than long-term ex-smokers (1.96%), though statistical comparisons were limited by the single case (n=1) in the long-term ex-smoker group. No statistically significant differences were observed between long-term ex-smokers and current smokers (p= 0.5791) or ex-smokers (<10 years, p=0.6198), nor between long-term ex-smokers and never smokers (p=0.5690).

### Multivariable analysis of pneumonia risk

After adjusting for potential confounders, including age, sex, education level, residence, income, vaccination status, obesity, and chronic conditions (cardiovascular diseases, diabetes, chronic respiratory diseases, and malignant tumors), smoking status was significantly associated with pneumonia (Table 3). Compared to never smokers, Ex-smokers (<10 years) had the highest odds of developing pneumonia (AOR=3.48; 95% CI: 2.96–4.09), followed by current smokers (AOR=3.18; 95% CI: 2.90–3.48). No statistically significant difference was observed between long-term ex-smokers and never smokers (AOR=1.12; 95% CI: 0.45–2.41). In contrast, long-term ex-smokers (≥10 years) had a significantly lower risk compared to ex-smokers <10 years.

**Table 3:** Multivariable logistic regression analysis of adjusted associations between smoking status and COVID-19-related pneumonia from a nationwide cross-sectional online survey in China, Oct 2022–Feb 2023 (N=22709)

*Variables*	*Categories*	*AOR (95% CI)*
**Smoking status**	Never smokers ®	1
	Long-term ex-smokers (≥10 years)	1.12 (0.45–2.41)
	Ex-smokers <10 years	3.48 (2.96–4.09)
	Current smokers	3.18 (2.90–3.48)
**Sex**	Female ®	1
	Male	1.70 (1.55–1.86)
**Residence**	Rural ®	1
	Urban	1.14 (1.04–1.25)
**Education level**	Middle school or lower ®	1
	High school/vocational	0.80 (0.71–0.91)
	Bachelor’s or higher	0.72 (0.64–0.81)
**Income level** (RMB)	≥10000 ®	1
	6000–9999	1.16 (0.97–1.40)
	3000–5999	1.41 (1.18–1.68)
	1000–2999	1.66 (1.38–2.00)
	<1000	1.14 (0.91–1.43)
**Vaccination status**	Unvaccinated ®	1
	Vaccinated	1.11 (0.98–1.26)
**BMI** (kg/m^2^)	<30 ®	1
	≥30	1.23 (1.01–1.50)
**Chronic conditions**	No cardiovascular disease ®	1
	Cardiovascular disease	2.21 (2.00–2.43)
	No diabetes ®	1
	Diabetes	3.43(3.10–3.79)
	No chronic respiratory disease ®	1
	Chronic respiratory disease	2.54 (2.30–2.81)
	No malignant tumor ®	1
	Malignant tumor	1.63 (1.34–1.97)
**Age** (years)		1.001 (0.997–1.004)

AOR: adjusted odds ratio; models adjusted for age, sex, residence, education level, income, vaccination status, obesity, and chronic conditions. Pneumonia: defined as individuals who sought medical attention due to a COVID-19 infection and received a diagnosis of pneumonia during the medical visit. RMB: 1000 Chinese Renminbi about US$140. ® Reference categories.

The adjusted absolute risks of pneumonia by smoking status are presented in Table 4. From the multivariable logistic regression analysis, the highest estimated absolute risk of pneumonia was observed among ex-smokers (<10 years) at 22.42% (95% CI: 19.90–25.17%). This was followed by current smokers, with an adjusted absolute risk of 20.85% (95% CI: 19.66–22.09). In contrast, long-term former smokers (≥10 years) exhibited a markedly lower adjusted absolute risk of 8.51% (95% CI: 3.91–17.52), which was comparable to that of never smokers, who had the lowest adjusted absolute risk at 7.66% (95% CI: 7.25–8.10).

**Table 4:** Multivariable logistic regression analysis of adjusted absolute risk of COVID-19-related pneumonia according to smoking status from a nationwide cross-sectional online survey in China, Oct 2022–Feb 2023 (N=22709)

*Smoking status*	*Absolute* *risk*	*Lower* *95% CI*	*Upper* *95% CI*
Current smokers	0.2085	0.1966	0.2209
Ex-smokers <10 years	0.2242	0.199	0.2517
Long-term ex-smokers (≥10 years)	0.0851	0.0391	0.1752
Never smokers	0.0766	0.0725	0.0810

Models adjusted for age, sex, residence, education level, income, vaccination status, obesity, and chronic conditions. Pneumonia: defined as individuals who sought medical attention due to a COVID-19 infection and received a diagnosis of pneumonia during the medical visit.

For complete transparency, the detailed logistic regression results for hospitalization and severe COVID-19 outcomes are presented in Supplementary file Tables S1 and S2, respectively. Additionally, the logistic regression results for the analysis with combined ex-smoker categories are provided in Supplementary file Table S3.

### Sensitivity analyses

To assess the robustness of our findings, we conducted a sensitivity analysis that included all participants who self-reported COVID-19 infection, regardless of NAAT or SARS-CoV-2 Antigen Testing, yielding a total sample size of 59180. The association between smoking status and pneumonia remained consistent with the primary analysis, as shown in Supplementary file Table S4. Compared with never smokers, current smokers had an AOR of 3.22 (95% CI: 3.04–3.41), ex-smokers (<10 years) a 3.15 (95% CI: 2.82–3.52), and long-term ex-smokers (≥10 years) a 1.31 (95% CI: 0.78–2.08).

## DISCUSSION

This nationwide cross-sectional study investigated the association between smoking status and COVID-19 outcomes, with a novel focus on long-term smoking cessation and its relationship with the risk of pneumonia following COVID-19 infection. Our findings indicate that long-term ex-smokers (≥10 years) exhibited a marked reduction in pneumonia risk compared to current smokers and ex-smokers <10 years, with no statistically significant difference compared to never smokers, highlighting the potential benefits of long-term smoking cessation. By contrast, ex-smokers <10 years and current smokers had significantly higher odds of developing pneumonia compared to never smokers, with both groups demonstrating comparable levels of risk.

Although not enough studies have directly examined the association between long-term smoking cessation and pneumonia, our findings align with broader evidence on the benefits of long-term smoking cessation. A study by Choi^19^ reported that after adjusting for potential confounding factors, current smokers and recent ex-smokers were associated with an increased risk of death from COVID-19, while long-term ex-smokers and never smokers had a reduced risk of death from COVID-19 compared to current smokers. Additionally, pooled analyses from four national cohorts showed that individuals who quit smoking for ≥10 years had survival outcomes comparable to those of never smokers^17^.

Our results align with evidence suggesting that smoking may exacerbate adverse COVID-19 outcomes^5,20-22^, such as pneumonia, hospitalization, and severe COVID-19. However, heterogeneity exists within the literature. Some studies have reported no significant association between current smoking and COVID-19 outcomes^23^, and others have suggested a paradoxical protective effect of current smoking against COVID-19 outcomes^24,25^. One possible explanation for these inconsistencies lies in the health characteristics of individuals who quit smoking. A significant proportion of individuals quit smoking primarily due to health concerns^26^, people who had a chronic condition diagnosis was associated with intention to quit smoking^27^. Consistent with this, our results showed that the prevalence of chronic conditions was higher among ex-smokers compared to current smokers. These underlying health conditions can significantly influence the outcomes of COVID-19^28^.

The possible mechanisms linking associations between smoking and infection are structural changes in the respiratory tract and a decrease in immune response^29^, which increase the risk of infections, including COVID-19. Smoking influences both innate and adaptive immune responses. Its impact on innate immunity diminishes rapidly after smoking cessation, whereas the effects on adaptive responses persist long after quitting^30^. Notably, a systematic review observed individuals exposed to smoke have a decrease in antibody levels and avidity, and in immune cell production. The meta-analysis demonstrated significantly lower vaccine responses among smokers compared to non-smokers across a range of vaccines, including those against COVID-19, influenza, pneumococcus, with a weighted mean difference of 0.65 (95% CI: 0.10–1.19; p=0.02)^31^. These findings suggest that smokers are not only more vulnerable to infections but may also have diminished vaccine efficacy.

In addition to immune dysfunction, smoking accelerates lung function decline, further increasing susceptibility to respiratory infections. Smoking cessation, however, reverses some of these effects. For instance, individuals who quit smoking experience a slower rate of lung function decline and, in some cases, improved pulmonary function^32^. A prospective randomized clinical trial among smokers with mildto-moderate airway obstruction found that those who stopped smoking experienced an average improvement in FEV1 of 47 mL (or 2%) in the first year after quitting^33^.

In the context of COVID-19, the angiotensin-converting enzyme 2 (ACE2) receptor might play a critical role. ACE2 has been identified as the functional receptor of severe acute respiratory syndrome coronavirus 2 (SARS-CoV-2)^34^. Smoking-induced overexpression of ACE2 in human bronchial and alveolar epithelial cells might increase the risk of infection and disease severity^35^. Decreased ACE2 expression was observed in bronchial epithelial cells from ex-smokers compared with current smokers, especially in long-term ex-smokers^34^. These findings underscore the potential biological basis for the protective effects of long-term smoking cessation.

From a public health perspective, both current and former smokers may represent populations at elevated risk for respiratory infections, including but not limited to COVID-19. Ex-smokers often have a disproportionately high burden of chronic conditions, which may heighten their susceptibility to severe respiratory infections^7,36^. Therefore, identifying this group for enhanced monitoring or preventive strategies may be appropriate. While causal inferences cannot be drawn from the cross-sectional study, these patterns merit consideration in future longitudinal studies exploring targeted prevention strategies.

Future longitudinal studies with larger sample sizes and objective measures of smoking status and health outcomes are warranted to corroborate and expand upon our findings. Further longitudinal research is essential to elucidate the temporal effects of smoking cessation on respiratory infection risk. Understanding how the risk of respiratory infections evolves over time after smoking cessation is crucial for informing public health strategies.

### Limitations

Several limitations of our study should be acknowledged. First, the cross-sectional design precludes causal inference. Reverse causality may be a concern, particularly for individuals who recently quit smoking, as underlying health conditions could have motivated cessation. However, for long-term ex-smokers (≥10 years), the temporal separation between cessation and COVID-19 infection makes reverse causality unlikely. Second, the cross-sectional design and reliance on self-reported smoking status and COVID-19 outcomes may introduce recall and measurement bias. To mitigate this issue, we excluded patients who were infected before October 2022 to reduce potential recall inaccuracies. Additionally, missing data on smoking cessation were minimal (n=23; 0.1%). Third, the possibility of residual confounding cannot be excluded. Although major sociodemographic and clinical variables were adjusted for, unmeasured factors –such as ethnicity – may still influence COVID-19 outcomes. Nevertheless, given the high ethnic homogeneity in China (with over 90% of the population identifying as Han Chinese), the potential impact of race is likely limited. Finally, the number of long-term ex-smokers was relatively small, leading to wider confidence intervals for this group. Despite this limitation, long-term ex-smokers (≥10 years) continued to demonstrate a significantly lower risk of pneumonia compared to ex-smokers (<10 years), even after adjusting for confounding factors. This finding lends credibility to our observations. While our study provides valuable insights into the impact of long-term smoking cessation on COVID-19-related pneumonia, these limitations underscore the need for caution when interpreting these findings and assessing their generalizability, given the online, self-reported sample in China.

## CONCLUSIONS

In this nationwide cross-sectional online survey in China, long-term smoking cessation (≥10 years) was not associated with an increased risk of COVID-19-related pneumonia compared with never smokers, whereas ex-smokers (<10 years) appeared to remain at elevated risk. These findings support the potential benefits of sustained smoking cessation and highlight recent ex-smokers as a population that may warrant closer monitoring in the context of respiratory infections. Further longitudinal studies are needed to assess the evolution of risk following cessation and to explore appropriate preventive strategies.

## Supplementary Material



## Data Availability

The data supporting this research are available from the authors on reasonable request.
