# Supplementary material for: Association between long-term smoking cessation and COVID-19 outcomes: Findings from a nationwide crosssectional online survey in China
Source: Tob Induc Dis. 2025 Sep 26;23:10.18332/tid/209212. doi: 10.18332/tid/209212 (PMC12465114; doi:10.18332/tid/209212)
Supplement: Supplementary file 1 [file TID-23-141-s1.pdf]

Table S1. Adjusted Associations Between Smoking Status and Hospitalization from a Nationwide Cross-Sectional Online Survey in China (Oct 2022–Feb 2023; N=22,709): Multivariable Logistic Regression Analysis

Variable	Group	OR (95% CI)
**Smoking status**	Never smoker (ref)	
	Long-term ex-smoker (≥10 years)	0.66(0.11-2.15)
	Ex-smokers <10 years	1.58(1.23-2.00)
	Current smoker	1.63(1.43-1.86)
**Sex**	Female (ref)	-
	Male	1.20(1.06-1.36)
**Residence**	Rural (ref)	-
	Urban	0.89(0.79-1.01)
**Education**	Middle school and below (ref)	-
	High school/vocational	0.97(0.82-1.14)
	Bachelor’s or higher	0.66(0.57-0.78)
**Income level**	≥10000 (ref)	-
	6000-9999	0.80(0.64-1.00)
	3000-5999	0.74(0.60-0.92)
	1000-2999	0.70(0.56-0.89)
	<1000	0.80(0.61-1.05)
**Vaccination status**	Unvaccinated (ref)	—
	Vaccinated	0.53(0.46-0.61)
**Obesity**	BMI<30 (ref)	—
	BMI≥30	1.05(0.79-1.36)
**Chronic conditions**	No cardiovascular disease (ref)	—
	Cardiovascular disease	2.07(1.81-2.36)
	No diabetes (ref)	—
	Diabetes	2.16(1.88-2.48)
	No chronic respiratory disease (ref)	—
	Chronic respiratory disease	1.61(1.40-1.85)
	No malignant tumor (ref)	—

	Malignant tumor	1.15(0.86-1.51)
**Age (per year)**	Continuous variable	0.998(0.993-1.003)

Table S2. Adjusted Associations Between Smoking Status and Severe COVID-19 from a Nationwide Cross-Sectional Online Survey in China (Oct 2022–Feb 2023; N=22,709): Multivariable Logistic Regression Analysis

Variable	Group	OR (95% CI)
**Smoking status**	Never smoker (ref)	
	Long-term ex-smoker (≥10 years)	1.18(0.07-5.58)
	Ex-smokers <10 years	1.70(1.10-2.53)
	Current smoker	1.98(1.57-2.50)
**Sex**	Female (ref)	-
	Male	1.27(1.02-1.59)
**Residence**	Rural (ref)	-
	Urban	1.06(0.84-1.34)
**Education**	Middle school and below (ref)	-
	High school/vocational	0.91(0.69-1.20)
	Bachelor's or higher	0.56(0.43-0.73)
**Income level**	≥10000 (ref)	-
	6000-9999	1.26(0.81-2.04)
	3000-5999	1.02(0.67-1.63)
	1000-2999	1.12(0.72-1.83)
	<1000	1.05(0.62-1.83)
**Vaccination status**	Unvaccinated (ref)	—
	Vaccinated	0.55(0.43-0.72)
**Obesity**	BMI<30 (ref)	—
	BMI≥30	1.04(0.63-1.62)
**Chronic conditions**	No cardiovascular disease (ref)	—
	Cardiovascular disease	3.33(2.68-4.13)
	No diabetes (ref)	—
	Diabetes	2.31(1.82-2.91)

	No chronic respiratory disease (ref)	—
	Chronic respiratory disease	1.83(1.44-2.30)
	No malignant tumor (ref)	—
	Malignant tumor	1.15(0.71-1.76)
**Age (per year)**	Continuous variable	1.003(0.995-1.012)

Table S3. Associations Between Smoking Status and Pneumonia from a Nationwide Cross-Sectional Online Survey in China (Oct 2022–Feb 2023; N=22,709): Sensitivity Analysis with Ex-smokers Not Stratified by Cessation Duration

Variable	Group	OR (95% CI)
**Smoking status**	Never smoker (ref)	
	Ex-smokers	3.32(2.83-3.89)
	Current smoker	3.18(2.90-3.48)
**Sex**	Female (ref)	-
	Male	1.69(1.55-1.86)
**Residence**	Rural (ref)	-
	Urban	1.14(1.04-1.25)
**Education**	Middle school and below (ref)	-
	High school/vocational	0.80(0.71-0.91)
	Bachelor's or higher	0.72(0.64-0.81)
**Income level**	≥10000 (ref)	-
	6000-9999	1.17(0.97-1.40)
	3000-5999	1.41(1.18-1.68)
	1000-2999	1.66(1.38-2.00)
	<1000	1.14(0.91-1.43)
**Vaccination status**	Unvaccinated (ref)	—
	Vaccinated	1.11(0.98-1.26)
**Obesity**	BMI<30 (ref)	—
	BMI≥30	1.23(1.01-1.49)
**Chronic conditions**	No cardiovascular disease (ref)	—
	Cardiovascular disease	2.21(2.00-2.43)
	No diabetes (ref)	—

	Diabetes	3.44(3.11-3.80)
	No chronic respiratory disease (ref)	—
	Chronic respiratory disease	2.55(2.31-2.81)
	No malignant tumor (ref)	—
	Malignant tumor	1.62(1.33-1.97)
**Age (per year)**	Continuous variable	1.001(0.997-1.004)

Table S4. Sensitivity Analysis of the Adjusted Associations Between Smoking Status and COVID-19 Outcomes, including all participants with self-reported infection, from a Nationwide Cross-Sectional Online Survey in China (Oct 2022–Feb 2023; N=59,180)[1]

**COVID-19 Outcomes**	**Smoking status**	**OR (95% CI)**
Pneumonia[2]	Never smoker (ref)	
	Long-term ex-smoker (≥10 years)	1.31(0.78-2.08)
	Ex-smokers <10 years	3.15(2.82-3.52)
	Current smoker	3.22(3.04-3.41)
Hospitalization[3]	Never smoker (ref)	
	Long-term ex-smoker (≥10 years)	1.37(0.61-2.64)
	Ex-smokers <10 years	1.95(1.64-2.31)
	Current smoker	1.74(1.59-1.91)
Severe COVID-19[4]	Never smoker (ref)	
	Long-term ex-smoker (≥10 years)	1.78(0.43-4.77)
	Ex-smokers <10 years	2.42(1.81-3.19)
	Current smoker	2.35(2.00-2.75)

1. Sensitivity analysis includes all participants who self-reported COVID-19 infection, irrespective of NAAT or SARS-CoV-2 Antigen Testing results (N=59,180). Models adjusted for age, sex, residence, education, income, vaccination status, obesity, and chronic conditions.
2. Pneumonia: Defined as individuals who sought medical attention due to a COVID-19 infection and received a diagnosis of pneumonia during the medical visit.
3. Hospitalization: Defined as individuals who sought medical attention at healthcare facilities due to COVID-19 and required hospital admission.
4. Severe COVID-19: Defined as individuals who sought medical attention due to a COVID-19 infection and reported being diagnosed as severe or critical.

© 2025 Zhou X. et al.
